# Stand and Landscape Drivers of Trap Captures of the Pine Wilt Disease Vector *Monochamus alternatus* (Coleoptera: Cerambycidae) in the Qinling Mountains

**DOI:** 10.1002/ece3.74503

**Published:** 2026-10-11

**Authors:** Jingyu Qi, Xiang Zhang, Mengqin Zhao, Xiaomeng Gao, Chen Liang, Jaime C. Piñero, Kailang Yang, Hong He

**Affiliations:** ^1^ Key Laboratory of National Forestry and Grassland Administration for Control of Forest Biological Disasters in Western China, College of Forestry Northwest A&F University Yangling China; ^2^ Stockbridge School of Agriculture University of Massachusetts Amherst Massachusetts USA

**Keywords:** forest pests, landscape ecology, pine wilt disease, spatial scale, stand structure

## Abstract

*Monochamus alternatus* (Coleoptera: Cerambycidae) is the principal vector of pine wilt disease, one of the most destructive forest diseases affecting conifer ecosystems in East Asia. However, the relative roles of stand structure and surrounding landscape context in shaping spatial variation in trap captures of this vector in mountainous forests remain poorly understood. The associations of stand‐ and landscape‐level factors with trap captures of *M. alternatus* were assessed in the Qinling Mountains of central China, a topographically complex region experiencing rapid expansion of pine wilt disease. Twenty pine forest plots were monitored with pheromone traps from April to October 2023, and stand characteristics and landscape variables were quantified at multiple spatial scales. Generalized linear models and multimodel inference showed that the proportion of *Pinus massoniana* within stands was significantly positively associated with beetle trap captures, whereas the proportion of construction land within a 250 m radius was strongly negatively associated with beetle trap captures (*p* = 0.006, ∑wi = 1). Stand and landscape predictors contributed comparably to explaining spatial variation in trap captures. These results indicate that local host composition and fine‐scale anthropogenic disturbance are associated with spatial variation in *M. alternatus* adult activity in mountainous forest landscapes. The identification of 250 m as the spatial scale of strongest landscape association provides a spatially explicit reference for improving surveillance and management strategies for pine wilt disease in topographically complex forest systems.

## Graphical abstract

This study examines how stand structure and surrounding landscape context influence the trap‐based abundance of *Monochamus alternatus*, the principal vector of pine wilt disease, in the Qinling Mountains of China. Using pheromone‐trap monitoring, multi‐scale landscape metrics, and multimodel inference, we show that local host composition and fine‐scale anthropogenic disturbance jointly shape beetle abundance. These findings provide spatially explicit evidence to improve vector surveillance and forest pest management in mountainous pine forests.

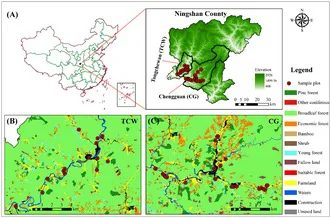

## 1 Introduction

Forest insect pests pose major threats to forest biodiversity, productivity, and ecosystem services, particularly under the combined pressures of climate change and intensifying human disturbance (Seidl et al. 2017; Pureswaran et al. 2018). These stressors may alter pest development, survival, and dispersal, as well as host tree physiology, thereby reshaping pest–host interactions across spatial and temporal scales. Because forest pest populations are embedded within heterogeneous forest landscapes, their abundance and outbreak risk are often regulated by both local stand conditions and broader landscape context. Population dynamics of forest pests are jointly influenced by local stand attributes such as tree density, canopy structure, and host composition, and by landscape‐level characteristics including habitat diversity, connectivity, and fragmentation (Li 2007; Lundquist and Hamelin 2009; Krasnov et al. 2019). Stand variables such as mean diameter at breast height (DBH), tree height, pine density, and the proportion of pine trees reflect stand development, host availability, and resource concentration, which may affect host selection, oviposition, and larval development, and local beetle activity in pine‐associated insects such as *Monochamus alternatus* (Jactel et al. 2021; Nan et al. 2023). Topographic variables, including elevation, slope, and aspect, may further influence beetle populations by shaping local temperature, humidity, solar radiation, host distribution, and seasonal activity patterns (Jung et al. 2024). Despite growing recognition that both local habitat conditions and landscape structure influence forest pest dynamics, their relative roles and spatial scales of influence may differ across forest ecosystems. In mountainous pine ecosystems, where steep topographic gradients and heterogeneous land‐use patterns create complex habitat mosaics, how stand conditions and surrounding landscapes are jointly associated with spatial variation in *M. alternatus* trap captures remains unclear. For example, Yang (2020) demonstrated that both stand and landscape variables regulate the abundance of *Dryocoetes* spp. (Coleoptera: Curculionidae), Yu et al. (2022) identified landscape edge density and forest cover as key predictors of *Tomicus* spp. (Coleoptera: Curculionidae) outbreaks in southwestern China, and Li et al. (2020) found that the density of *M. alternatus* Hope (Coleoptera: Cerambycidae) in Fujian Province was strongly affected by forest patch size, road density, and farmland proportion. Together, these findings suggest that forest pest abundance should be understood within a multiscale ecological framework that integrates local host and stand conditions with surrounding landscape composition and configuration. *Monochamus alternatus* is an important forest wood‐boring pest and the principal vector of the pinewood nematode *Bursaphelenchus xylophilus* (Steiner & Buhrer) in East Asia. Pine wilt disease (PWD), caused by this nematode, is characterized by rapid transmission, high host mortality, and limited management options (Togashi and Arakawa 2003). Since its first detection in Nanjing in 1982, PWD has expanded to 638 counties across 17 provinces in China (Announcement No. 4, 2025, Shaanxi Forestry Administration), resulting in economic losses exceeding 100 billion RMB and placing more than 60 million ha of pine forests at risk (State Forestry Administration of China 2024) (Ye 2019).

The Qinling Mountains constitute a major biogeographic boundary between temperate and subtropical forest zones in central China. Their steep elevational gradients and complex terrain create heterogeneous environmental conditions and diverse pine forest habitats (Yao et al. 2020). These landscapes support widespread occurrence of *M. alternatus* but are also experiencing one of the country's most rapid expansions of PWD. Since PWD was first detected in the region in 2009, it has affected nearly 100,000 ha of pine forest, resulting in the death of more than 50 million trees and direct economic losses exceeding 25 billion RMB. In response, an additional 7 million trees have been preventively removed to slow its spread, incurring further losses of more than 3 billion RMB (Announcement No. 4, 2025, Shaanxi Forestry Administration). The combination of diverse pine habitats, severe disease pressure, and heterogeneous landscape structure makes the Qinling Mountains an ideal system for examining how local stand conditions and surrounding landscape characteristics jointly relate to *M. alternatus* trap captures in mountainous environments. Because the efficiency of PWD transmission is closely linked to the population density and dispersal behavior of its insect vector (Togashi and Arakawa 2003), identifying the spatial factors associated with *M. alternatus* adult activity is important for vector surveillance and disease management.

Previous landscape‐ecology studies have shown that host‐tree distribution, forest fragmentation, landscape configuration, and environmental conditions can strongly influence the abundance, movement, and population dynamics of forest insects, including bark beetles and wood‐boring beetles (Sanguansub et al. 2012; Hong et al. 2018; Økland et al. 2019; Yu et al. 2022). Although many of these studies focus on insect outbreaks, the same landscape processes are also relevant to vector‐mediated pathogen spread, because host availability, habitat connectivity, and landscape heterogeneity can affect vector beetle activity and host encounters. However, similar analyses remain scarce in mountainous regions of central China, where complex topography and diverse land‐use patterns generate highly heterogeneous landscapes. The Qinling Mountains therefore provide a valuable opportunity to examine whether associations between host distribution, landscape fragmentation, and land‐use heterogeneity and *M. alternatus* activity vary across spatial scales (Huang et al. 2016; Yao et al. 2020). Such scale‐dependent landscape effects may be less apparent in more homogeneous low‐elevation pine systems. Despite growing recognition that both local habitat conditions and landscape structure influence forest insect dynamics, few studies have simultaneously evaluated stand‐ and landscape‐level associations with *M. alternatus* activity across multiple spatial scales in mountainous pine ecosystems. In particular, how topographically complex landscapes modify the relationship between stand‐level host availability and landscape‐level habitat configuration remains unclear. The associations of stand‐ and landscape‐level factors with trap captures of *M. alternatus* were examined in Ningshan County, a mountainous region of the Qinling Mountains. Specifically, three questions were addressed: (1) How do *M. alternatus* trap captures vary among pine forest plots during the adult activity season? (2) Which stand‐ and landscape‐level factors best explain spatial variation in beetle captures? (3) At which spatial scale are landscape variables most strongly associated with beetle trap captures? By addressing these questions, this study aimed to improve understanding of the stand‐ and landscape‐level correlates of spatial variation in *M. alternatus* trap captures in topographically complex mountainous forests and provide ecological evidence for more spatially targeted pine wilt disease surveillance and management.

## 2 Materials and Methods

### 2.1 Study Area and Sampling Design

This study was conducted in Ningshan County, Ankang City, Shaanxi Province, on the southern slope of the Qinling Mountains in central China (33°07′11″–33°50′38″ N, 108°02′33″–108°56′48″ E). The county covers an area of 3678.5 km^2^ and is characterized by complex mountainous terrain, with elevations ranging from 400 to 2825 m. The region has a humid subtropical climate with a mean annual temperature of 12.5°C. Forests cover approximately 96% of the county area (Table S1). The dominant vegetation type is secondary forest, including mixed conifer–broadleaf stands and secondary stands containing pine species. Within these forests, *Pinus tabuliformis* , *P. armandii* , and *P. massoniana* occupy approximately 18,666 ha and occur in both pine‐dominated and mixed stands, serving as host trees for *M. alternatus* . The pine‐containing stands selected for this study were generally middle‐aged, with typical stand ages of approximately 50–60 years, and exhibited a multilayered structure consisting of an overstory tree layer, a shrub layer and an herbaceous understory. Such variation in stand structure and host composition creates heterogeneous habitat conditions for *M. alternatus* .

Two PWD‐affected townships, Chengguan (CG) and Tongchewan (TCW), were selected because they contain large areas of secondary forest with pine species, have documented occurrence of pine wilt disease, and represent typical mountainous forest landscapes in Ningshan County. Because suitable pine stands in this region are naturally distributed across complex terrain and because traps had to be repeatedly accessed throughout the adult flight season, the study followed an observational field design rather than an experimentally stratified landscape design. Site selection was therefore constrained by the availability of pine stands, accessibility, safety, and the feasibility of sustained monitoring (Figure 1A).

**FIGURE 1:**
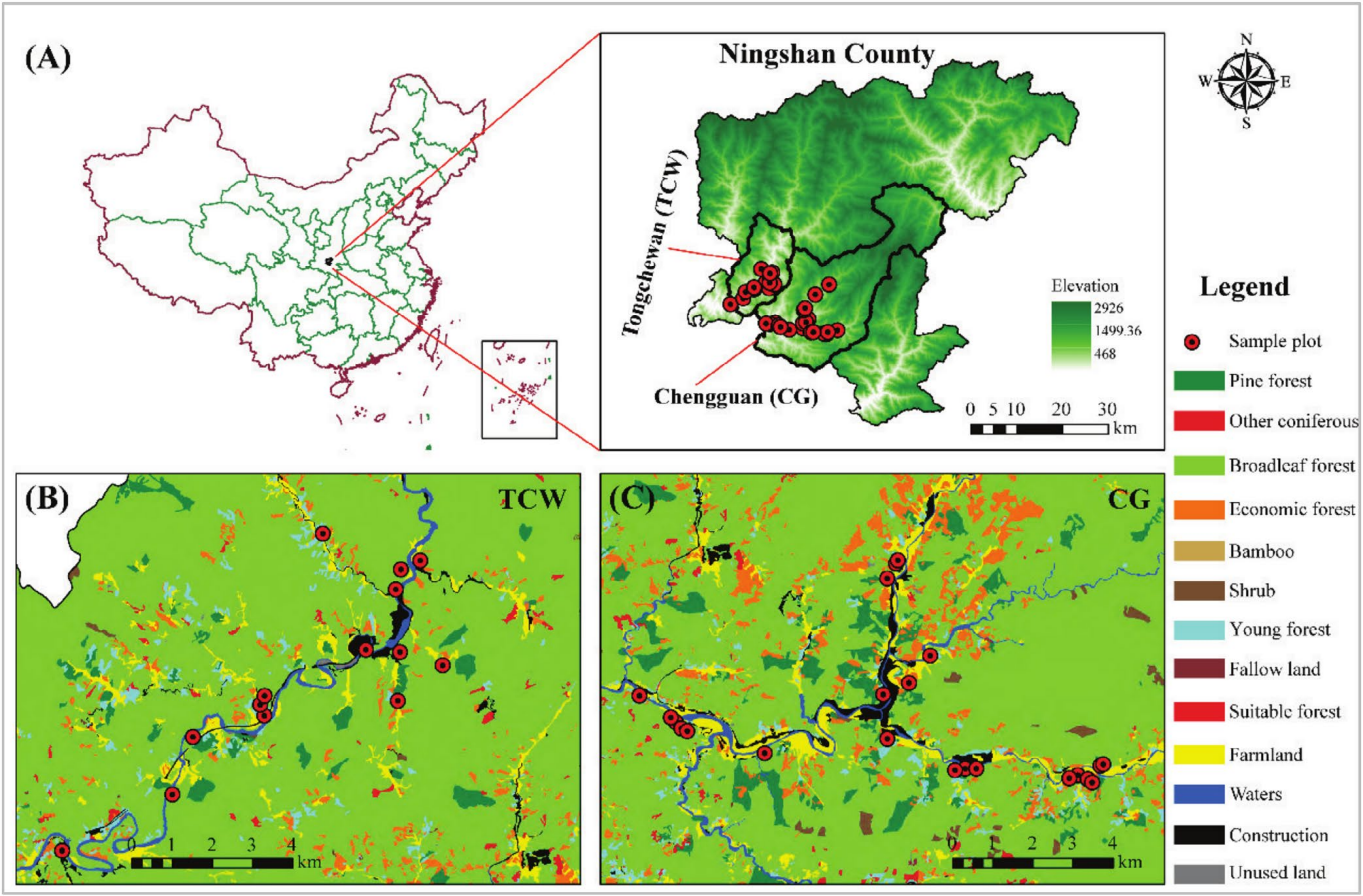
Locations of sampling plots and landscape types in Ningshan County, Qinling Mountains, China. (A) Location of the study area and distribution of sampling plots in Tongchewan (TCW) and Chengguan (CG) townships. (B, C) Distribution of sampling plots and landscape types in the TCW and CG regions, showing 13 classified land‐cover types.

In April 2023, 20 sampling sites were established across these two townships, with 10 sites in each township (Table S2; Figure 1B,C). To the extent possible, sites were selected to capture variation in pine host composition, stand structure, elevation, and surrounding land‐use context while maintaining spatial separation among sampling locations. At each site, a 20 m × 20 m plot was used to characterize local stand structure and pine host species. Geographic coordinates of each plot center were recorded using a handheld GPS. The spatial distribution of sampling plots and landscape types is shown in Figure 1. Adult *M. alternatus* were captured from April to October 2023 using ZM‐60 cross‐vane traps (Chen Kang Agroforestry, Fujian, China) baited with F8 lure (Hangzhou Pheromone Biotechnology Co. Ltd.). The lure was supplied as a two‐component slow‐release system, with the host volatiles contained in a polyethylene slow‐release bottle and the aggregation pheromone in a polyethylene slow‐release sachet. The surfaces of the cross vanes and the inner surface of the funnel were treated with a manufacturer‐applied efficiency‐enhancing coating. At each site, three traps were deployed at relatively open and well‐ventilated locations within or around the pine forest, with traps spaced at least 100 m apart and the trap bottom positioned at least 1.5 m above the ground. Traps were suspended from pine branches using iron wires, with the lure placed inside the trap according to the manufacturer's instructions. Trap catches were checked weekly to record beetle captures and seasonal activity, and lures were replaced every 45 days to maintain trapping efficiency.

### 2.2 Stand Variables, Landscape Classification and Landscape Metrics

The Forest Resources Management “One Map” dataset for Ningshan County was processed in ArcGIS 10.8 to characterize the landscape surrounding each sampling site. Land cover types were grouped into forested and non‐forested categories according to the original land‐use classification (Table S2). Forested land included pine forest, other coniferous forest, broadleaf forest, economic forest (tree forest in Table S2), bamboo, shrub, young forest stands, fallow land, and land suitable for afforestation. Non‐forested land included farmland, water, construction, and unused land. Landscape variables were calculated within circular buffers of 250, 500, 750, and 1000 m around each sampling site to characterize landscape composition and configuration at multiple spatial scales. All spatial vector layers were converted to raster format at a 20‐m resolution in ArcGIS 10.8 and subsequently imported into FRAGSTATS 4.2 for landscape metrics analysis (McGarigal et al. 2023). Stand variables were measured only within the forested sampling plots, whereas landscape variables were calculated based on all land‐cover types within the defined analysis extent, including both forested and non‐forested land. Eight stand variables and three landscape variables were included in the statistical analysis (Table 1). Because dead or visibly declining pine trees were routinely removed as part of local pine wilt disease control, no dead pine trees were observed within the sampling plots during the field survey. Accordingly, dead‐tree abundance was not included as a stand variable. To characterize landscape structure, a broader set of landscape metrics was initially calculated in FRAGSTATS 4.2. At the class level, these included number of patches (NP), class area (CA), patch density (PD), percentage of landscape (PLAND), largest patch index (LPI), mean patch area (AREA_MN), edge density (ED), landscape shape index (LSI), and aggregation index (AI) (Table S3). At the landscape level, total area (TA), NP, LPI, contagion index (CONTAG), splitting index (SPLIT), Shannon diversity index (SHDI), Shannon evenness index (SHEI), Simpson's diversity index (SIDI), and AI were calculated (Table S4). These metrics were used to characterize landscape composition, fragmentation, configuration, and heterogeneity.

**TABLE 1:** Stand and landscape variables used in the analysis of *Monochamus alternatus* trap captures, with definitions, calculation methods, and data ranges.

Category	Variable	Description	Calculation/data source	Data range
Stand variables	Mean DBH (cm)	Mean diameter at breast height of pine trees	Arithmetic mean of pine trees with DBH ≥ 2 cm measured in the plot	9.29 to 28.73
	Height (m)	Mean height of pine trees	Arithmetic mean of measured pine individuals within the plot	8 to 22
	Pine density (trees/ha^−1^)	Density of living and recently cut host pines	Number of living pine trees and recently cut pine stumps divided by plot area and converted to ha^−1^	0.03 to 0.38
	Elevation (m)	Elevation of the sampling plot	Recorded at the plot center using a handheld GPS	634 to 1046
	Slope (°)	Mean slope within the plot	Measured in the field within each plot	16 to 42
	Southwestness	Southwest orientation of slope aspect	Calculated as the cosine of the deviation of aspect from 225°	−1 to 1
	Canopy	Proportion of the plot covered by the tree canopy	Measured in the field within each sampling plot	0.35 to 0.80
	Pine proportion (%)	Proportion of pine trees within the plot	Number of pine trees divided by the total number of trees × 100	0 to 100
Landscape variables	Construction (%)	Proportion of construction land	PLAND of construction land within each buffer, calculated in FRAGSTATS 4.2	0 to 100
	Cropland (%)	Proportion of cropland	PLAND of cropland within each buffer, calculated in FRAGSTATS 4.2	0 to 100
	SIDI	Simpson's diversity index; overall landscape heterogeneity	Calculated from all land‐cover classes within each buffer using FRAGSTATS	0 to 1

Among these metrics, the landscape variables used in the statistical analyses were selected to represent the composition and heterogeneity of the landscape surrounding each sampling site. The proportions of construction land and cropland, calculated using the percentage of landscape metric (PLAND), together with SIDI, were carried forward as FRAGSTATS‐derived predictors in the statistical analyses. These three variables were calculated separately within 250‐, 500‐, 750‐, and 1000‐m buffers around each sampling site. In addition, distance from buildings (DFB) and distance from roads (DFR) were calculated in ArcGIS 10.8 as the Euclidean distances from the center of each sampling site to the nearest building and road, respectively, as indicators of local human disturbance. The stand‐ and landscape‐level variables used in the statistical analyses, together with their definitions, calculation methods, units, and data ranges, are summarized in Table 1.

### 2.3 Stand Structure and Scale Analysis

All trees with DBH ≥ 2 cm were measured in each plot to characterize overall stand structure. This threshold was not intended to define trees suitable for *M. alternatus* infestation. Eight stand variables were considered: mean DBH, tree height, pine density (trees ha^−1^), elevation, slope, southwestness, canopy, and pine proportion. Mean DBH and tree height were calculated from measured pine individuals within each plot. Pine density was calculated as the number of living pine trees per unit area and expressed as trees ha^−1^ (Park et al. 2013). Stumps from earlier years were excluded. Recently cut stumps were included because they may still provide suitable resources for *M. alternatus* development. Slope aspect was quantified using the southwestness index, defined as the cosine of the deviation from 225° (Yu et al. 2023). The index ranges from −1 to 1, with higher positive values indicating slopes more closely oriented toward the southwest. Canopy was recorded as the percentage of canopy cover within each plot. For the three pine species, the proportion of each species was calculated as the number of individuals of that species divided by the total number of trees within the plot × 100.

To evaluate the spatial scale at which surrounding landscape characteristics were associated with local *M. alternatus* trap captures (He 2013), circular buffers with radii of 250, 500, 750, and 1000 m were delineated around each sampling site (Zhang et al. 2007). These buffer distances were not intended to represent the maximum flight distance of individual beetles (Ma et al. 2018) but to examine landscape associations across multiple spatial scales. The buffers were overlaid with the classified land‐cover map, and landscape variables were calculated separately for each site and spatial scale.

### 2.4 Statistical Analysis

The associations of stand‐ and landscape‐level factors with *M. alternatus* trap captures were assessed using generalized linear models (GLMs) within a multimodel inference framework. The response variable was the cumulative number of adult beetles captured per plot during the monitoring period, standardized by trapping effort. Trap captures were treated as an index of relative adult activity among sampling sites rather than a direct estimate of absolute population density. Because the response variable consisted of count data and showed evidence of overdispersion, GLMs were fitted using a negative binomial distribution with a log link function. Trap‐days were included as a log‐transformed offset to standardize sampling effort among plots.

At the stand level, candidate models were constructed using combinations of structural and topographic variables, including mean DBH, tree height, pine density, proportion of pine trees, elevation, slope, and aspect. At the landscape level, candidate models were developed separately for four spatial extents (250, 500, 750, and 1000 m) based on landscape variables quantified within each buffer. The optimal spatial scale was determined by comparing candidate models using corrected Akaike information criterion (AICc), with the spatial extent yielding the lowest AICc value selected for subsequent analyses. For each candidate model set, model selection was conducted using AICc. Models with ΔAICc < 4 were considered to have sufficient support and were included in model averaging. Model averaging was conducted using the full averaging approach, and the relative importance of each predictor was evaluated based on cumulative Akaike weights (∑wi). Variables retained from the stand‐ and landscape‐level analyses were subsequently combined to construct an integrated model to evaluate their relative contributions when considered jointly.

Prior to model construction, Spearman's rank correlation analysis was used to assess pairwise correlations among explanatory variables. Variables showing strong correlations (|r| > 0.7) were not included simultaneously in the same candidate model to reduce multicollinearity. Variance inflation factors (VIFs) were further calculated to confirm that multicollinearity was not substantial (VIF < 4). To reduce model complexity, variables showing weak associations with trap captures (|r| < 0.2) were excluded before model selection.

All analyses were conducted in R version 4.3.3 using the MuMIn package for multimodel inference (Bartoń 2023; R Core Team 2024), MASS for negative binomial modeling (Venables and Ripley 2002), and car for VIF computation (Fox and Weisberg 2019).

## 3 Results

### 3.1 Seasonal Variation in Adult *M. alternatus* Activity

Adults of *M. alternatus* recorded a seasonal pattern across the 20 sampling sites (Figure 2). Beetles were first captured in April, and trap counts increased gradually, reaching the first peak between May and June, followed by a decline in July. At a few sites, captures increased again after July, reaching a second peak in August before declining sharply in September. Most plots exhibited a single annual peak, whereas clear bimodal patterns were observed at five sites: Site 1: XFC, Site 2: LJG, Site 4: RHH, Site 10: LWTV, and Site 20: YWV (Figure 2). Several individual plots showed a single peak occurring in May, June, July, or August, suggesting local variation in seasonal activity patterns among sites.

**FIGURE 2:**
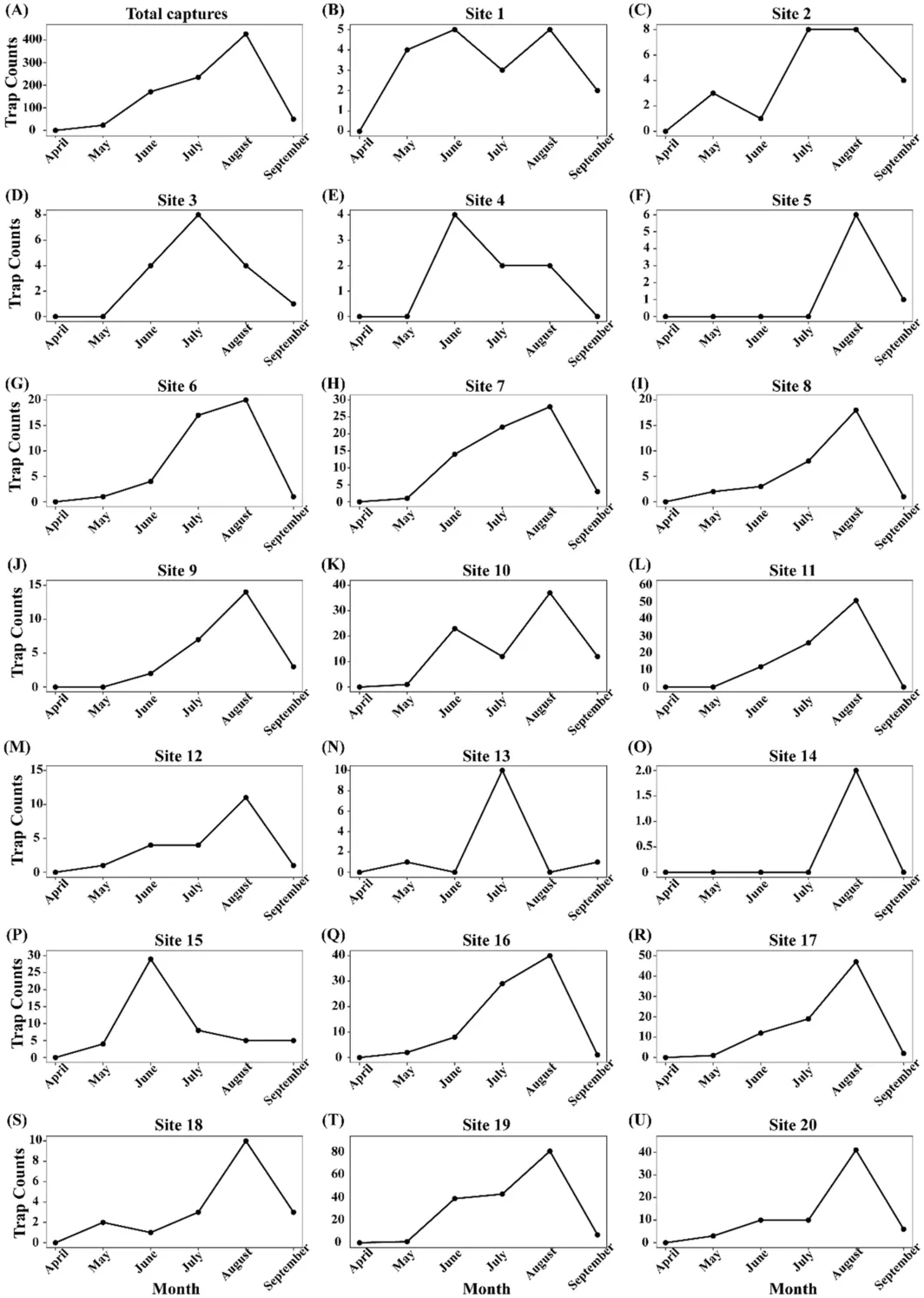
Seasonal dynamics of *Monochamus alternatus* trap captures across 20 sampling sites in Ningshan County. (A) Monthly total captures summed across all sampling sites. (B–U) Monthly trap captures at individual sampling sites: Xingfu Community (B), Liujia Gully (C), Qinjia Bay (D), Rear Hill of Hospital (E), Shaojia Courtyard (F), Haitangyuan Village (G), Hanba Village (H), Huayan Village (I), Longquan Village (J), Longwangtan Village (K), Banqiao (L), Tangping Village (M), Huachang (N), Qiligoukou (O), Yinyang Gully (P), Yaoshu Ridge (Q), Angou (R), Dahua Ping (S), Youfangping Village (T), and Yuwan Village (U).

### 3.2 Effects of Stand‐Level Factors on *M. alternatus* Captures

At the stand level, Spearman correlation analysis revealed no significant association between *M. alternatus* trap captures and stand density, mean DBH, canopy cover, or slope aspect (|r| < 0.2) (Figure 3). Because strong collinearity was detected among the proportions of the three pine species (|r| > 0.7), only the proportion of *P. massoniana* was retained for subsequent analyses. Elevation, slope, and *P. massoniana* proportion exhibited low mutual collinearity (|r| < 0.7) and were therefore included in the final model.

**FIGURE 3:**
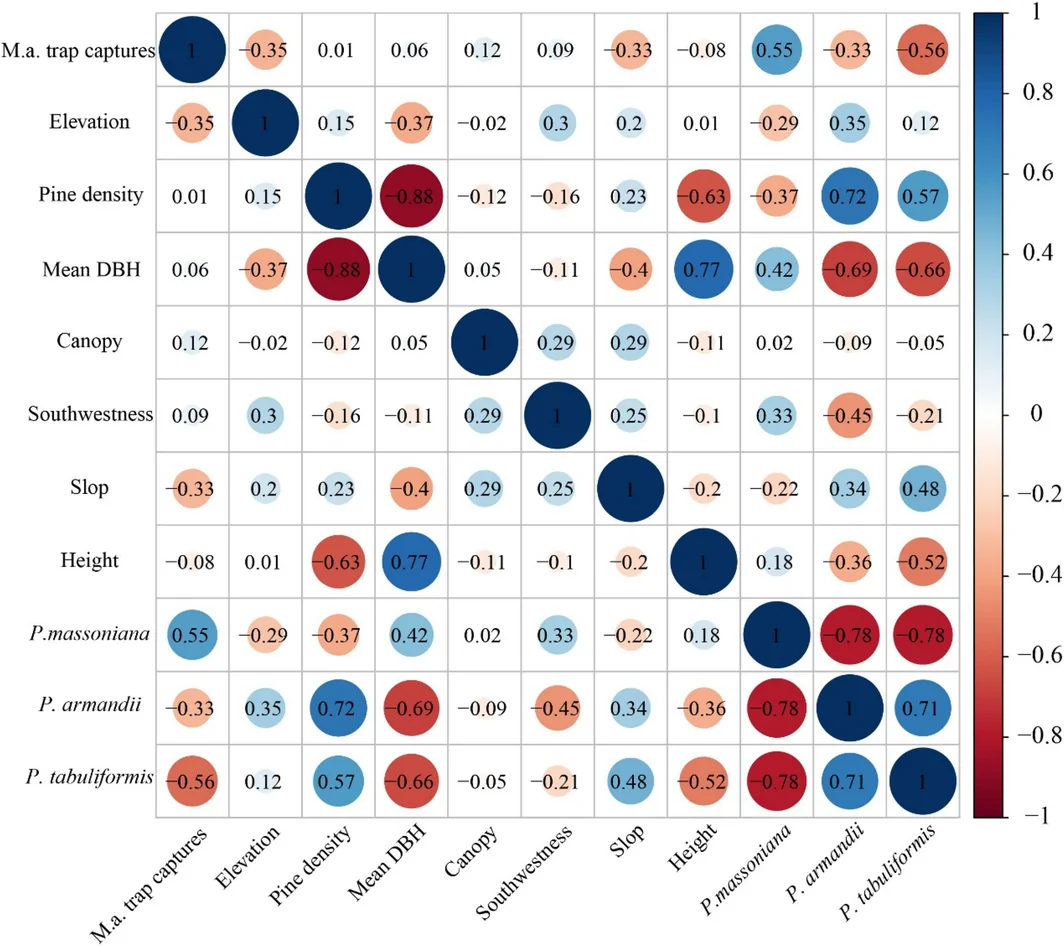
Spearman correlations between stand variables and *Monochamus alternatus* trap captures. *M. alternatus* captures represent the cumulative number of adults recorded per sampling plot.

Model averaging indicated that *P. massoniana* proportion was significantly and positively associated with *M. alternatus* trap captures (*p* = 0.023, relative importance = 0.89), whereas neither slope (*p* = 0.350) nor elevation (*p* = 0.448) had a significant effect (Figure 4A, Table 2).

**FIGURE 4:**
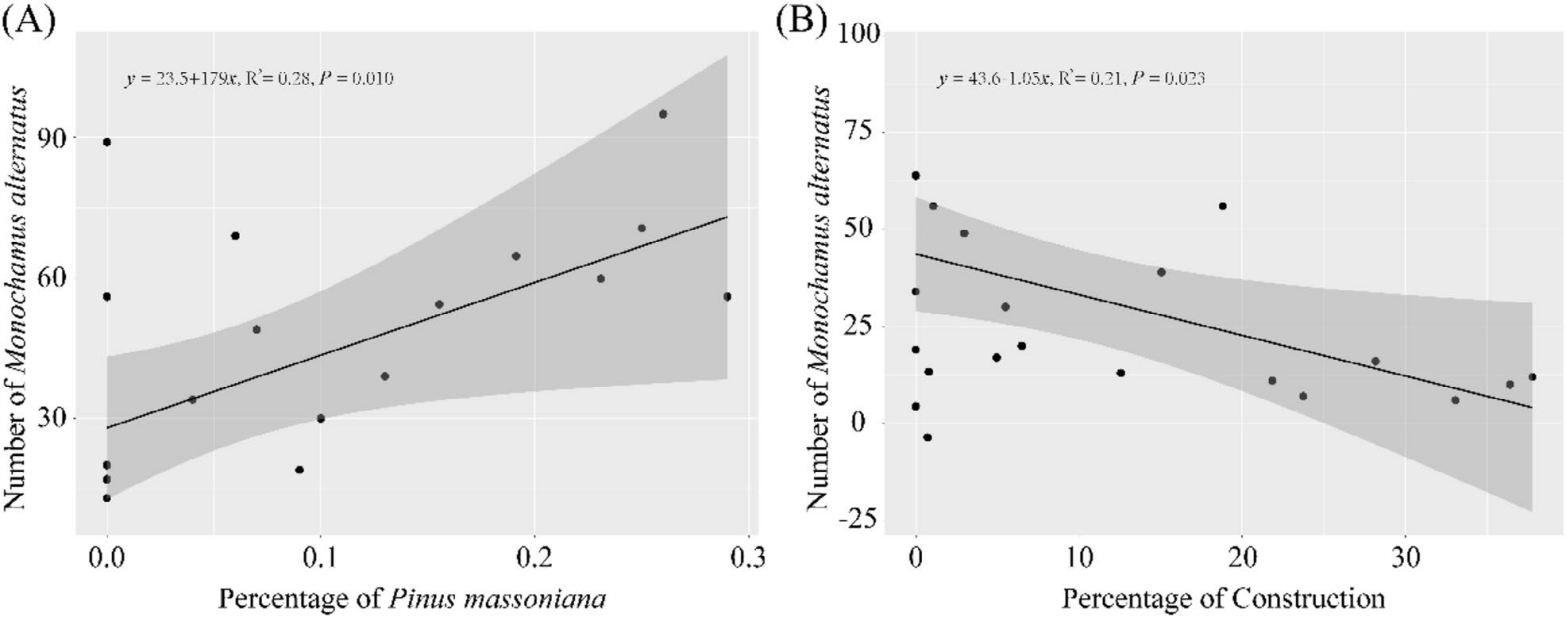
Relationships between stand and landscape variables and *Monochamus alternatus* trap captures: (A) proportion of *Pinus massoniana* within stands; (B) proportion of construction land in the surrounding landscape.

**TABLE 2:** Model‐averaged estimates showing the effects of stand variables on *Monochamus alternatus* trap captures.

Variables	Estimate	Std. error	*z*	Pr (> |z|)	Importance
Intercept	2.10	2.18	0.91	0.365	
Proportion of *P. massoniana*	2.09	0.84	2.28	0.023*	0.89
Slope	−0.29	0.28	0.94	0.350	0.18
Elevation	−0.56	0.67	0.76	0.448	0.15

*Note:* Pr (> |z|) denotes the two‐tailed *p‐*value associated with the *z*‐statistic of the model‐averaged coefficient.

* *p* < 0.05.

### 3.3 Effects of Landscape‐Level Factors on *M. alternatus* Captures

Among the tested spatial scales, the model with a 250‐m buffer had the lowest AICc value and accounted for 60.5% of the Akaike weight (Table 3). Models at broader scales (500 m and 1000 m) had higher AICc values, indicating weaker support. Within the best‐supported model set (ΔAICc < 4), the proportion of construction area was the only variable that consistently showed a significant negative association with *M. alternatus* trap captures (*p* = 0.006, relative importance = 1; Figure 4B, Table 4). Other landscape variables, including cropland proportion and forest diversity, had low importance values (< 0.4) and were not significantly associated with trap captures.

**TABLE 3:** Model selection for landscape‐level predictors of *M. alternatus* trap captures across spatial scales (ΔAICc < 4).

Landscape scale	Model	df	logLik	AICc	ΔAICc	Weight
250 m	**Construction**	**3**	**−3.217**	**13.9**	**0**	**0.605**
	Construction + SIDI	4	−3.058	16.8	2.85	0.145
	Construction + Cropland	4	−3.217	17.1	3.17	0.124
500 m	Construction	3	−5.143	17.8	0	0.431
	SIDI	3	−6.022	19.5	1.76	0.179
	Null	2	−7.767	20.2	2.45	0.126
	Construction + SIDI	4	−5.117	20.9	3.12	0.091
	Construction + Cropland	4	−5.128	20.9	3.14	0.09
750 m	Construction	3	−4.73	17	0	0.32
	Construction + Cropland	4	−3.323	17.3	0.35	0.268
	Construction + SIDI	4	−3.763	18.2	1.23	0.172
	Null	2	−7.767	20.2	3.28	0.062
	Construction + Cropland + SIDI	5	−2.995	20.3	3.32	0.061
	Cropland	3	−6.515	20.5	3.57	0.054
1000 m	Construction + Cropland	4	−2.658	16	0	0.337
	Construction	3	−4.326	16.2	0.17	0.309
	Construction + SIDI	4	−3.438	17.5	1.56	0.154
	Construction + Cropland + SIDI	5	−2.518	19.3	3.34	0.063
	Cropland + SIDI	4	−4.616	19.9	3.92	0.048

*Note:* Bold values indicate the best model. “Construction” refers to the proportion of construction, “Cropland” to the proportion of farmland, and “SIDI” to the Simpson's Diversity Index of the landscape.

**TABLE 4:** Model‐averaged estimates and relative importance of variables for competitive models (ΔAICc < 4).

Scale	Variables	Estimate	Std. error	*z*	Pr (> |z|)	Importance
250 m	Intercept	1.64	0.24	6.55	< 2e‐16	
	**Construction**	**−0.27**	**0.09**	**2.76**	**0.006** **	**1**
	SIDI	−0.57	1.10	0.48	0.629	0.17
	Cropland	0.00	0.10	0.01	0.996	0.14
500 m	Intercept	1.80	0.66	2.60	0.009	
	Construction	−0.17	0.09	1.78	0.075	0.67
	SIDI	−1.62	2.68	0.58	0.565	0.29
	Cropland	−0.02	0.11	0.15	0.883	0.1
750 m	Intercept	1.28	0.58	2.12	0.034	
	**Construction**	**−0.20**	**0.10**	**2.00**	**0.046** *	**0.88**
	Cropland	0.22	0.15	1.39	0.164	0.41
	SIDI	2.35	2.09	1.05	0.296	0.25
1000 m	Intercept	1.31	0.56	2.24	0.025	
	**Construction**	**−0.20**	**0.09**	**2.12**	**0.034** *	**0.95**
	Cropland	0.26	0.16	1.55	0.122	0.49
	SIDI	1.18	2.64	0.43	0.667	0.29

*Note:* Bold type indicates statistically significant predictor variables at *p* < 0.05. Construction = percentage of construction land; Cropland = percentage of cropland. Pr (> |z|) denotes the two‐tailed *p‐*value associated with the *z*‐statistic of the model‐averaged coefficient.

Abbreviation: SIDI, Simpson's diversity index.

* *p* < 0.05.

** *p* < 0.01.

### 3.4 Integrated Multilevel Model

Significant variables identified at the stand and landscape scales were combined into an integrated model. Spearman correlation and VIF analyses indicated no strong collinearity among predictors (|r| < 0.7, VIF < 4; Figure 5, Table 5). Distance from roads showed no notable association with *M. alternatus* trap captures (|r| < 0.2) and was therefore excluded from the final model.

**FIGURE 5:**
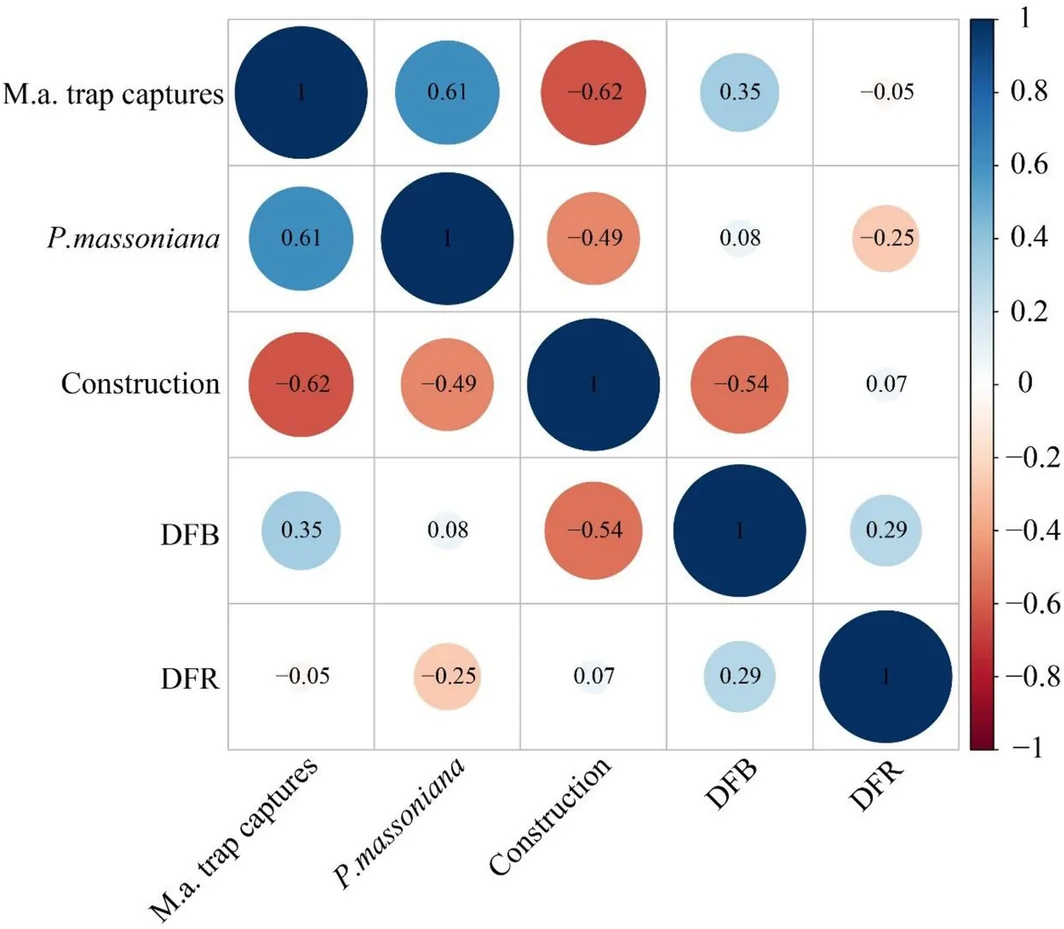
Spearman correlation matrix showing relationships among stand‐ and landscape‐level variables and *Monochamus alternatus* trap captures. DFB = distance from buildings; DFR = distance from roads.

**TABLE 5:** Variance inflation factors (VIF) for predictors included in the integrated model.

Variable	Percentage of *P. massoniana*	Construction	DFB
VIF	1.06	1.65	1.57

Abbreviation: DFB, distance from buildings.

Model averaging based on the candidate models containing the selected stand‐ and landscape‐scale variables identified *P. massoniana* proportion and construction land proportion as the most important predictors of *M. alternatus* trap captures (Table 6). Both showed significant effects (*p* = 0.009 and *p* = 0.008, respectively), with relative importance values of 1.

**TABLE 6:** Model‐averaged estimates of the effects of stand‐ and landscape‐level variables on *Monochamus alternatus* trap captures.

Variables	Estimate	Std. error	*z*	Pr (> |z|)	Importance
Intercept	1.47	0.18	7.57	< 0.001	
**Construction**	**−0.13**	**0.04**	**2.64**	< 0.01**	**1**
**Proportion of *P. massoniana* **	**2.20**	**0.78**	**2.62**	< 0.01**	**1**
DFB	0.02	0.07	0.24	0.814	0.15

*Note:* Bold type indicates statistically significant predictor variables (*p* < 0.05). Double asterisks (**) indicate significance at *p* < 0.01. The intercept is excluded from the significance marking convention.

Abbreviation: DFB, distance from buildings.

## 4 Discussion

### 4.1 Stand‐Level Effects on *M. alternatus* Trap Captures

This study examined how stand‐ and landscape‐level factors are associated with trap captures of *M. alternatus* in the Qinling Mountains. Previous studies have shown that the abundance of *M. alternatus* is influenced by multiple stand characteristics, although the relative importance of these factors varies among regions. For example, Yao et al. (2008) reported that vegetation cover, slope aspect, and forest location significantly influenced beetle abundance in the Ma'anshan forests of Anhui Province. Zhu (2009) identified elevation, slope position, DBH, tree height, and stand structure as key determinants in Wanding, Yunnan, whereas Jiang et al. (2020) found that beetle abundance in low‐elevation forests of Huangshan was closely related to stand age, canopy density, slope position, and species mixture ratio. These differences suggest that regional variation in *M. alternatus* abundance may be associated with differences in the measured stand and topographic attributes, particularly pine host species, stand development, elevation, and slope position. In our study, the proportion of *P. massoniana* within stands emerged as an important local driver, indicating that host availability may be especially relevant for explaining spatial variation in beetle trap captures in the Qinling Mountains.

The pronounced topographic complexity of the Qinling Mountains may create strong local heterogeneity in forest composition, host availability, and microclimatic conditions. Such heterogeneity can influence adult activity, dispersal, and population distribution, thereby contributing to spatial variation in *M. alternatus* adult activity among sites. Consequently, regional differences in beetle captures may reflect ecological responses to heterogeneous mountain environments rather than stochastic variation alone (Seidl et al. 2018). This highlights the importance of considering fine‐scale environmental and landscape context when evaluating vector populations and potential pine wilt disease risk in mountainous forests.

At the stand level, only the proportion of *P. massoniana* was positively associated with *M. alternatus* trap captures, suggesting that pine host species composition provided greater explanatory support for variation in trap captures than general stand structure. In contrast, the negative correlations with *P. tabuliformis* and *P. armandii* may partly reflect the lower relative availability of *P. massoniana* in stands dominated by these species, rather than indicating that these pine species are unsuitable hosts. This result is consistent with the known host preference of *M. alternatus* . In the Qinling–Daba Mountains, *P. massoniana* has been reported as a preferred food source for adult supplementary feeding, although oviposition preference and larval density may differ among pine species (Nan et al. 2023). Therefore, the positive association observed in our study should be interpreted primarily as a response of adult activity and trap captures to host composition. In addition, host chemical cues may partly explain this pattern. *P. massoniana* releases abundant monoterpenes such as (+)‐α‐pinene, (−)‐α‐pinene, and limonene following feeding damage, which function as key volatile cues mediating host location (Wen et al. 2024). Such volatile compounds may influence host location and feeding behavior of adult beetles. In contrast, the other measured stand variables, including mean DBH, tree height, pine density, elevation, slope, and aspect, were not strong predictors in the present analysis. This may indicate that, within our study plots, adult trap catches were more closely related to host species composition than to overall stand size, stand density, or topographic position. Mean DBH and tree height may reflect stand development and host biomass, but they do not necessarily capture host suitability or adult attraction when pine species composition varies among plots. Similarly, pine density alone may be less informative than the proportion of the preferred host species. Topographic variables may influence beetle activity indirectly through microclimate and host distribution, but their effects may have been weak or masked by local host composition in this study (Stage and Salas 2007). Together, these results suggest that *P. massoniana* ‐dominated stands may represent areas of elevated adult *M. alternatus* activity and may warrant particular attention in vector surveillance.

### 4.2 Seasonal Variation in Adult Activity

Seasonal patterns of trap capture also varied among sites. Most plots exhibited a single peak in adult activity, whereas a subset of sites showed a bimodal pattern with a second peak in August, suggesting that local stand conditions, host availability, topography, and surrounding land‐use context may contribute to spatial variation in adult activity (Kwon et al. 2019). Similar variability has been reported in other regions; for example, populations in eastern, western, southern, and central Guangxi have been reported to exhibit one to three activity peaks depending on local environmental conditions and host availability (Zheng et al. 2023). Elevation, slope, and aspect can alter local temperature, humidity, and solar radiation (Jia et al. 2024), thereby influencing adult emergence, flight activity, and trap captures. In mountainous terrain, additional fine‐scale features such as valleys, ravines, and proximity to streams may further create cooler or more humid microhabitats (McNichol et al. 2024), potentially delaying or extending adult activity at some sites. Future studies combining pheromone trapping with microclimatic monitoring and repeated annual surveys would help determine whether bimodal activity patterns reflect variation in emergence timing, host condition, local population density, or movement among nearby pine patches.

### 4.3 Landscape‐Level Effects and Implications

Landscape composition also played an important role in explaining variation in beetle trap captures. Numerous studies have shown that outbreaks and dispersal of forest insects are strongly influenced by landscape structure (Thierry et al. 2022; Blank 2025). For example, landscape‐scale connectivity has been linked to outbreak dynamics of the Eurasian spruce bark beetle *Ips typographus* Linnaeus (Coleoptera: Scolytidae) (Seidl et al. 2016), and landscape configuration influences the spread of Yunnan shoot beetles (*Tomicus yunnanensis* and *Tomicus minor*) (Coleoptera: Scolytidae) in pine forests (Yu et al. 2022). In this study, the proportion of construction land within a 250 m radius showed a strong negative association with *M. alternatus* trap captures. This finding is consistent with recent work showing that the proportion of buildings influenced trap catches of bark‐ and wood‐boring beetles, indicating that built environments can affect beetle captures and should be considered when interpreting trapping data (Nardi et al. 2026). However, the 250 m radius should not be interpreted as the maximum dispersal distance of *M. alternatus* . Adults of this species can disperse over hundreds of meters to several kilometers, and occasional long‐distance movement or human‐mediated transport may occur over much larger distances (Hu et al. 2013). Rather, the 250 m radius identified in our analysis likely represents the fine‐scale landscape context most closely associated with local trap catches, host availability, and adult activity around each sampling site.

Constructed areas in this study are therefore unlikely to function as absolute physical barriers to *M. alternatus* movement (Larroque et al. 2022). If suitable pine stands occur beyond this distance, adults may still disperse across or around built‐up areas. Instead, a high proportion of construction land within the immediate surroundings of traps may represent a local non‐host or highly disturbed matrix that reduces the continuity of pine host patches, weakens habitat connectivity, and may be associated with reduced beetle activity near traps. This interpretation is consistent with landscape‐genetic evidence showing that host and mixed forests promote gene flow in *M. alternatus* , whereas farmland and urban land uses are negatively associated with gene flow and may suppress spread (Han et al. 2023). Therefore, the 250‐m scale should be viewed as the strongest local landscape association detected in our trap‐based analysis, while broader spatial scales may still be important for dispersal, population connectivity, and pine wilt disease spread. The 250‐m spatial scale identified in this study may provide a useful reference for understanding the local landscape context associated with *M. alternatus* trap captures. This fine‐scale landscape context may therefore warrant particular attention when designing future monitoring programs in fragmented mountainous forests. The non‐significant effects of most landscape variables suggest that fine‐scale host and land‐use conditions may be more relevant than broader landscape configuration indices in explaining *M. alternatus* captures in the Qinling Mountain landscape. Several factors may explain these non‐significant results. First, *M. alternatus* is strongly dependent on pine hosts for adult feeding, oviposition, and larval development, and host composition within stands may override weaker landscape‐level effects when suitable pine resources are locally available (Nan et al. 2023). Second, rugged terrain and heterogeneous forest mosaics may modify habitat distribution, local environmental conditions, and beetle movement pathways, resulting in spatially variable relationships between landscape characteristics and trap captures (Uuemaa et al. 2009). Third, trap catches mainly reflect local adult activity near sampling points, whereas some landscape variables may better represent broader dispersal or population connectivity processes. Finally, the relatively limited number of sampling plots may have reduced statistical power to detect weaker landscape effects, because the ability to identify small or moderate effects generally increases with sample size (Quinn and Keough 2002).

The sampling design also provides useful context for interpreting seasonal patterns in trap captures. Although the number of sampling plots was constrained by logistical limitations associated with intensive field surveys in rugged mountainous terrain, the plots were distributed across contrasting stand structures and landscape contexts and were monitored throughout the adult activity season. Nevertheless, the relatively small sample size may have limited our ability to detect weaker relationships. In addition, because the study covered a single year, interannual variation in *M. alternatus* activity could not be evaluated. The patterns identified here should therefore be interpreted in the context of the Qinling Mountains. Disease‐related variables may also influence *M. alternatus* activity. However, the density of *B. xylophilus*‐infected trees and disease severity were not systematically quantified during our field surveys and therefore could not be included in the analysis. In addition, dead or visibly declining pine trees were routinely removed as part of local pine wilt disease‐control practices, and no dead pine trees were observed within the sampling plots during the survey. Future studies incorporating additional environmental measurements, such as host‐tree condition, woody material availability, and fine‐scale habitat characteristics (Sukovata et al. 2023), together with more sampling plots and repeated annual surveys, would help clarify whether these seasonal differences reflect variation in emergence timing, population dynamics, host status, or movement among nearby pine patches.

Overall, our results indicate that *M. alternatus* trap captures in the Qinling Mountains were associated with both local host composition and surrounding landscape context. Trap captures were positively associated with the proportion of *P. massoniana* within stands and negatively associated with construction land within a 250 m radius, with 250 m representing the spatial scale of strongest observed landscape association. These findings highlight the potential value of considering both host composition and surrounding land‐use context when interpreting *M. alternatus* trap captures and designing future surveillance programs in mountainous pine forests.

## 5 Conclusion

This study shows that *M. alternatus* activity, expressed as trap captures, in the Qinling Mountains exhibits distinct seasonal patterns and is associated with both local host composition and fine‐scale landscape context. Adult captures generally increased from spring to early summer, with most sites showing a single annual peak and several sites exhibiting bimodal activity patterns, indicating spatial variation in seasonal adult activity. Across stand‐level predictors, the proportion of *P. massoniana* was the strongest positive predictor of beetle captures, whereas other stand structural variables showed limited explanatory power. At the landscape scale, construction land within 250 m was the strongest negative predictor, indicating that nearby land‐use context was associated with variation in *M. alternatus* trap captures. These findings show that variation in trap captures in mountainous pine forests cannot be explained by stand structure alone. Instead, both host composition within stands and fine‐scale surrounding landscape context should be considered when interpreting vector trap captures and evaluating pine wilt disease risk. These patterns may help inform future site‐specific monitoring and early‐warning strategies for *M. alternatus* in high‐relief forest regions.

## Author Contributions

**Jingyu Qi:** formal analysis (lead), supervision (equal), writing – original draft (lead). **Xiang Zhang:** data curation (equal), investigation (equal), methodology (equal). **Mengqin Zhao:** data curation (equal), investigation (equal), methodology (equal). **Xiaomeng Gao:** data curation (equal), investigation (equal). **Chen Liang:** investigation (equal). **Jaime C. Piñero:** methodology (equal), writing – review and editing (equal). **Kailang Yang:** methodology (equal), writing – review and editing (equal). **Hong He:** funding acquisition (equal), writing – review and editing (equal).

## Funding

This work was supported by The Forestry Science and Technology Innovation Project of Shaanxi Province (SXLK2021‐0101), Interdisciplinary Frontier Innovation Team Program of Northwest A&F University (A1080524002), and The Special Fund of Shaanxi Provincial Forest Pest Control and Quarantine Station (20191217000008, 20191217000009).

## Conflicts of Interest

The authors declare no conflicts of interest.

## Supporting information


**Table S1:** Landscape composition and patch metrics in Ningshan County.
**Table S2:** Geographic location and elevation of the 20 sampling sites in Chengguan and Tongchewan townships, Ningshan County.
**Table S3:** Patch metrics and landscape indices for different land‐cover types in Ningshan County.
**Table S4:** Landscape‐level metrics used to analyze variation in *Monochamus alternatus* abundance in Ningshan County.

## Data Availability

The data supporting the findings of this study are available in the Dryad Digital Repository at https://doi.org/10.5061/dryad.1ns1rn98d.

## References

[ece374503-bib-0001] Bartoń, K. 2023. “MuMIn: Multi‐Model Inference. R Package Version 1.47.5.” https://CRAN.R‐project.org/package=MuMIn.

[ece374503-bib-0002] Blank, L. 2025. “How Urban Proximity Shapes Agricultural Pest Dynamics: A Review.” Pest Management Science 81: 2704–2711. 10.1002/ps.8671. PMC12074629 39831377

[ece374503-bib-0003] Fox, J. , and S. Weisberg . 2019. An R Companion to Applied Regression. Sage. https://socialsciences.mcmaster.ca/jfox/Books/Companion/.

[ece374503-bib-0004] Han, X. H. , Y. C. Li , W. L. Huang , et al. 2023. “Landscapes Drive the Dispersal of *Monochamus alternatus*, Vector of the Pinewood Nematode, Revealed by Whole‐Genome Resequencing.” Forest Ecology and Management 529: 120682. 10.1016/j.foreco.2022.120682.

[ece374503-bib-0005] He, D. J. 2013. Landscape Ecology. China Forestry Publishing House.

[ece374503-bib-0006] Hong, C. , Z. Zhang , S. Cai , et al. 2018. “Analysis and Risk Prediction of Pinewood Nematodes Based on Landscape Patterns: A Case Study From Yichang, China.” Chinese Journal of Ecology 37: 3371–3380. 10.13292/j.1000-4890.201811.022.

[ece374503-bib-0007] Hu, S. J. , T. Ning , D. Y. Fu , et al. 2013. “Dispersal of the Japanese Pine Sawyer, *Monochamus alternatus* (Coleoptera: Cerambycidae), in China's Mainland as Inferred From Molecular Data and Associations to Indices of Human Activity.” PLoS One 8: e57568. 10.1371/journal.pone.0057568. PMC3585188 23469026

[ece374503-bib-0008] Huang, M. Y. , R. Y. Duan , S. X. Wang , Z. G. Wang , and W. Y. Fan . 2016. “Species Presence Frequency and Diversity in Different Patch Types Along an Altitudinal Gradient: *Larix chinensis* Beissn in Qinling Mountains, China.” PeerJ 4: e1803. 10.7717/peerj.1803. PMC4797764 26998409

[ece374503-bib-0009] Jactel, H. , X. Moreira , and B. Castagneyrol . 2021. “Tree Diversity and Forest Resistance to Insect Pests: Patterns, Mechanisms and Prospects.” Annual Review of Entomology 66: 277–296. 10.1146/annurev-ento-041720-075234. 32903046

[ece374503-bib-0010] Jia, J. L. , A. C. Hughes , M. H. Nunes , et al. 2024. “Forest Structural and Microclimatic Patterns Along an Elevational Gradient in Mount Kenya.” Agricultural and Forest Meteorology 356: 110188. 10.1016/j.agrformet.2024.110188.

[ece374503-bib-0011] Jiang, X. , Z. Wang , J. Yao , C. Xu , and Y. Li . 2020. “Correspondence Analysis of Quantitative Distribution of *Monochamus alternatus* and Environmental Factors at Middle and Low Elevation Area of Mount Huangshan.” Forest Pest and Disease 39: 16–19. 10.19688/j.cnki.issn1671-0886.20200003.

[ece374503-bib-0012] Jung, B. J. , M. G. Lee , and S. W. Kim . 2024. “Prediction of Potential Habitat of *Monochamus alternatus* Based on Shared Socioeconomic Pathway Scenarios.” Forests 15: 1563. 10.3390/f15091563.

[ece374503-bib-0013] Krasnov, H. , Y. Cohen , E. Goldshtein , et al. 2019. “The Effect of Local and Landscape Variables on Mediterranean Fruit Fly Dynamics in Citrus Orchards Utilizing the Ecoinformatics Approach.” Journal of Pest Science 92: 453–463. 10.1007/s10340-018-1023-8.

[ece374503-bib-0014] Kwon, S. H. , M. S. Go , J. Park , et al. 2019. “The Bimodal Adult Activity of *Monochamus alternatus* (Coleoptera: Cerambycidae) Caught in Pheromone Traps in Jeju Can be Explained by the Competitive Attractiveness of Dying Pine Trees.” Entomological Research 49: 172–178. 10.1111/1748-5967.12343.

[ece374503-bib-0015] Larroque, J. , J. Wittische , and P. M. A. James . 2022. “Quantifying and Predicting Population Connectivity of an Outbreaking Forest Insect Pest.” Landscape Ecology 37: 763–778. 10.1007/s10980-021-01382-9. PMC8897358 35273428

[ece374503-bib-0016] Li, C. D. 2007. Forest Entomology. China Forestry Publishing House.

[ece374503-bib-0017] Li, Y. C. , Y. J. Guo , X. Q. Weng , et al. 2020. “Influence of Landscape Patterns on Population Density of *Monochamus alternatus* (Coleoptera: Cerambycidae).” Scientia Silvae Sinica 56: 80–88.

[ece374503-bib-0018] Lundquist, J. E. , and R. C. Hamelin . 2009. Forest Pathology: From Genes to Landscape. APS Press.

[ece374503-bib-0019] Ma, T. , X. Shi , J. Shen , et al. 2018. “Field Evaluation of Commercial Attractants and Trap Placement for Monitoring Pine Sawyer Beetle, *Monochamus alternatus* (Coleoptera: Cerambycidae) in Guangdong, China.” Journal of Economic Entomology 111: 239–246. 10.1093/jee/tox281. 29186543

[ece374503-bib-0020] McGarigal, K. , S. A. Cushman , and E. Ene . 2023. “FRAGSTATS v4: Spatial Pattern Analysis Program for Categorical Maps.” https://www.fragstats.org.

[ece374503-bib-0021] McNichol, B. H. , R. Wang , A. Hefner , C. Helzer , S. M. McMahon , and S. E. Russo . 2024. “Topography‐Driven Microclimate Gradients Shape Forest Structure, Diversity, and Composition in a Temperate Refugial Forest.” Plant‐Environment Interactions 5: e10153. 10.1002/pei3.10153. PMC11166229 38863691

[ece374503-bib-0022] Nan, J. , J. Qi , Y. Yang , et al. 2023. “Population Density and Host Preference of the Japanese Pine Sawyer (*Monochamus alternatus*) in the Qinling–Daba Mountains of China.” Insects 14: 181. 10.3390/insects14020181. PMC9967673 36835750

[ece374503-bib-0023] Nardi, D. , D. Rassati , A. Battisti , et al. 2026. “Integrating Landscape Ecology Into Generic Surveillance Plans for Bark‐ and Wood‐Boring Beetles.” Ecological Applications 36: e70194. 10.1002/eap.70194. PMC12968951 41797626

[ece374503-bib-0024] Økland, B. , D. Flø , M. Schroeder , et al. 2019. “Range Expansion of the Small Spruce Bark Beetle *Ips amitinus*: A Newcomer in Northern Europe.” Agricultural and Forest Entomology 21: 286–298. 10.1111/afe.12331.

[ece374503-bib-0025] Park, Y. S. , Y. J. Chung , and Y. S. Moon . 2013. “Hazard Ratings of Pine Forests to a Pine Wilt Disease at Two Spatial Scales, Individual Trees and Stands, Using Self‐Organizing Map and Random Forest.” Ecological Informatics 13: 40–46. 10.1016/j.ecoinf.2012.10.008.

[ece374503-bib-0026] Pureswaran, D. S. , A. Roques , and A. Battisti . 2018. “Forest Insects and Climate Change.” Current Forestry Reports 4: 35–50. 10.1007/s40725-018-0075-6.

[ece374503-bib-0027] Quinn, G. P. , and M. J. Keough . 2002. Experimental Design and Data Analysis for Biologists. Cambridge University Press.

[ece374503-bib-0028] R Core Team . 2024. “R: A Language and Environment for Statistical Computing. R Foundation for Statistical Computing.” https://www.R‐project.org/.

[ece374503-bib-0029] Sanguansub, S. , H. Goto , and N. Kamata . 2012. “Guild Structure of Ambrosia Beetles Attacking a Deciduous Oak Tree *Quercus serrata* in Relation to Wood Oldness and Seasonality in Three Locations in Central Japan.” Entomological Science 15: 42–55. 10.1111/j.1479-8298.2011.00484.x.

[ece374503-bib-0030] Seidl, R. , K. Albrich , D. Thom , and W. Rammer . 2018. “Harnessing Landscape Heterogeneity for Managing Future Disturbance Risks in Forest Ecosystems.” Journal of Environmental Management 209: 46–56. 10.1016/j.jenvman.2017.12.014. PMC5873512 29275284

[ece374503-bib-0031] Seidl, R. , J. Müller , T. Hothorn , C. Bässler , M. Heurich , and M. Kautz . 2016. “Small Beetle, Large‐Scale Drivers: How Regional and Landscape Factors Affect Outbreaks of the European Spruce Bark Beetle.” Journal of Applied Ecology 53: 530–540. 10.1111/1365-2664.12540. PMC4816203 27041769

[ece374503-bib-0032] Seidl, R. , D. Thom , M. Kautz , et al. 2017. “Forest Disturbances Under Climate Change.” Nature Climate Change 7: 395–402. 10.1038/nclimate3303. PMC5572641 28861124

[ece374503-bib-0033] Stage, A. R. , and C. Salas . 2007. “Interactions of Elevation, Aspect, and Slope in Models of Forest Species Composition and Productivity.” Forest Science 53: 486–492. 10.1093/forestscience/53.4.486.

[ece374503-bib-0034] Sukovata, L. , T. Jaworski , A. Dziuk , A. Rutkiewicz , and R. Plewa . 2023. “Optimizing Trap Placement for Monitoring *Monochamus galloprovincialis* and Minimizing Bycatch of Beneficial Insects.” Sylwan 167: 804–827. 10.26202/sylwan.2023057.

[ece374503-bib-0035] Thierry, C. , B. Pisanu , and N. Machon . 2022. “Both Landscape and Local Factors Influence Plant and Hexapod Communities of Industrial Water‐Abstraction Sites.” Ecology and Evolution 12: e8365. 10.1002/ece3.8365. PMC8855018 35222941

[ece374503-bib-0036] Togashi, K. , and Y. Arakawa . 2003. “Horizontal Transmission of *Bursaphelenchus xylophilus* Between Sexes of *Monochamus alternatus* .” Journal of Nematology 35: 7–16. PMC2620615 19265968

[ece374503-bib-0037] Uuemaa, E. , M. Antrop , J. Roosaare , R. Marja , and Ü. Mander . 2009. “Landscape Metrics and Indices: An Overview of Their Use in Landscape Research.” Living Reviews in Landscape Research 3: 1–28. 10.12942/lrlr-2009-1.

[ece374503-bib-0038] Venables, W. N. , and B. D. Ripley . 2002. Modern Applied Statistics With S. 4th ed. Springer.

[ece374503-bib-0039] Wen, Q. M. , R. X. Chen , T. Xu , and D. J. Hao . 2024. “Functional Characterization of Terpene Synthases From Masson Pine ( *Pinus massoniana* ) Under Feeding of *Monochamus alternatus* Adults.” Forests 15: 244. 10.3390/f15020244.

[ece374503-bib-0040] Yang, L. 2020. Effects of Cropland Use Changes on Population Abundance of *Helicoverpa armigera* (Hübner): A Landscape Perspective Chinese Academy of Agricultural Sciences. 10.27630/d.cnki.gznky.2020.000042.

[ece374503-bib-0041] Yao, S. , L. Wang , C. Piao , and Q. Shu . 2008. “Influence of Forest Stand Factors on the Amount of *Monochamus alternatus* Hope.” Journal of Anhui Agricultural University 35: 411–415. 10.13610/j.cnki.1672-352x.2008.03.028.

[ece374503-bib-0042] Yao, Y. H. , Y. F. Hu , Z. X. Kou , and B. P. Zhang . 2020. “Spatial Patterns of *Pinus tabulaeformis* and *Pinus massoniana* Forests in Qinling‐Daba Mountains and the Boundary of Subtropical and Warm Temperate Zones.” Journal of Geographical Sciences 30: 1523–1533. 10.1007/s11442-020-1797-5.

[ece374503-bib-0043] Ye, J. 2019. “Epidemic Status of Pine Wilt Disease in China and Its Prevention and Control Techniques and Counter Measures.” Scientia Silvae Sinica 55: 1–10. 10.11707/j.1001-7488.20190901.

[ece374503-bib-0048] Yu, L. , Z. Zhan , L. Ren , H. Li , H. Huang , and Y. Luo . 2023. “Impact of Stand‐ and Landscape‐Level Variables on Pine Wilt Disease‐Caused Tree Mortality in Pine Forests.” Pest Management Science 79: 1791–1799. 10.1002/ps.7357. 36637393

[ece374503-bib-0044] Yu, L. , Z. Zhan , L. Ren , Y. Liu , H. Huang , and Y. Luo . 2022. “Effects of Stand and Landscape Level Variables on Shoot Damage Ratios Caused by Shoot Beetles in Southwest China.” Forest Ecology and Management 507: 120030. 10.1016/j.foreco.2022.120030.

[ece374503-bib-0045] Zhang, J. J. , R. Z. Zhang , and J. Y. Chen . 2007. “Species and Their Dispersal Ability of *Monochamus* as Vectors to Transmit *Bursaphelenchus xylophilus* .” Journal of Zhejiang A&F University 24: 350–356.

[ece374503-bib-0046] Zheng, G. N. , X. H. Yang , M. L. Wei , and X. L. Zheng . 2023. “Population Dynamics of *Monochamus alternatus* Adults in Guangxi and Its Correlation With Pine Stand and Meteorological Factors.” Scientia Silvae Sinica 59: 128–142. 10.11707/j.1001-7488.LYKX20210609.

[ece374503-bib-0047] Zhu, Y. P. 2009. Spatial Pattern of *Monochamus alternatus* Population Master's thesis, Northeast Forestry University.

