# Supplementary material for: Stand and Landscape Drivers of Trap Captures of the Pine Wilt Disease Vector Monochamus alternatus (Coleoptera: Cerambycidae) in the Qinling Mountains
Source: Ecol Evol. 2026 Oct 11;16(10):e74503. doi: 10.1002/ece3.74503 (PMC13654653; doi:10.1002/ece3.74503)
Supplement: Supplementary file 1 [file ECE3-16-e74503-s001.docx]

**TABLE S1** Landscape composition and patch metrics in Ningshan County.

| Type | Patch type | CA / hm^2^ | CA / % | NP | NP / % |
| --- | --- | --- | --- | --- | --- |
| Forest land  （96%） | Tree forest | 341997.40 | 93.26 | 407 | 4.65 |
|  | Bamboo | 17.67 | 0.01 | 21 | 0.24 |
|  | Shrub | 4297.60 | 1.17 | 344 | 3.93 |
|  | Young forest stands | 4548.89 | 1.24 | 2347 | 26.81 |
|  | Fallow land | 71.11 | 0.02 | 110 | 1.26 |
|  | land suitable for  afforestation | 1802.82 | 0.49 | 486 | 5.55 |
| Non-forest land  （4%） | Farmland | 7375.84 | 2.01 | 2458 | 28.08 |
|  | Water | 3115.28 | 0.85 | 129 | 1.47 |
|  | Construction | 1562.81 | 0.43 | 775 | 8.85 |
|  | Unused land | 1921.48 | 0.52 | 1677 | 19.16 |
| All | | 366710.90 | 100 | 8754 | 100 |

Note: CA, class area; NP, number of patches

**TABLE S2** Geographic location and elevation of the 20 sampling sites in Chengguan and Tongchewan townships, Ningshan County.

| Study location | Site | Longitude (E) | Latitude (N) | Altitude (m) |
| --- | --- | --- | --- | --- |
| CG | Xingfu Community (XFC) | 108.3101 | 33.3066 | 829.20 |
|  | Liujia Gully (LJG) | 108.3091 | 33.3165 | 816.87 |
|  | Qinjia Bay (QJB) | 108.2439 | 33.3164 | 784.94 |
|  | Rear Hill of Hospital (RHH) | 108.3339 | 33.2998 | 805.82 |
|  | Shaojia Courtyard (SJC) | 108.2522 | 33.3115 | 780.70 |
|  | Haitangyuan Village (HTYV) | 108.2272 | 33.4088 | 808.90 |
|  | Hanba Village (HBV) | 108.2338 | 33.4153 | 770.20 |
|  | Huayan Village (HYV) | 108.2397 | 33.3917 | 916.60 |
|  | Longquan Village (LQV) | 108.3678 | 33.3007 | 681.65 |
|  | Longwangtan Village (LWTV) | 108.1920 | 33.3805 | 686.11 |
| TCW | Banqiao (BQ) | 108.2077 | 33.4214 | 686.02 |
|  | Tangping Village (TPV) | 108.2316 | 33.3138 | 639.52 |
|  | Huachang (HC) | 108.3131 | 33.3467 | 688.80 |
|  | Qiligoukou (QLGK) | 108.2566 | 33.3084 | 898.36 |
|  | Yinyang Gully (YYG) | 108.2225 | 33.3142 | 687.16 |
|  | Yaoshu Ridge (YSR) | 108.3649 | 33.2966 | 721.59 |
|  | Angou (AG) | 108.2286 | 33.4133 | 798.36 |
|  | Dahua Ping (DHP) | 108.1728 | 33.3757 | 718.37 |
|  | Youfangping Village (YFPV) | 108.3680 | 33.3944 | 767.58 |
|  | Yuwan Village (YWV) | 108.3361 | 33.3739 | 666.24 |

**TABLE S3** Patch metrics and landscape indices for different land-cover types in Ningshan County.

| Patch type | CA | PLAND | LPI | NP | PD | AREA_MN | LSI | ED | AI |
| --- | --- | --- | --- | --- | --- | --- | --- | --- | --- |
| Pine forest | 37421.78 | 10.20 | 0.33 | 2227 | 0.61 | 16.80 | 84.59 | 17.81 | 95.68 |
| Other coniferous forest | 10308.52 | 2.81 | 0.44 | 437 | 0.12 | 23.59 | 34.05 | 3.73 | 96.74 |
| Broadleaf forest | 285266.05 | 77.79 | 69.95 | 869 | 0.24 | 328.27 | 69.42 | 39.32 | 98.72 |
| Economic forest | 8990.37 | 2.45 | 0.05 | 2369 | 0.65 | 3.80 | 74.03 | 7.63 | 92.29 |
| Bamboo | 19.97 | 0.01 | 0.01 | 23 | 0.01 | 0.87 | 7.00 | 0.03 | 86.17 |
| Shrub | 4297.60 | 1.17 | 0.10 | 344 | 0.09 | 12.49 | 31.80 | 2.20 | 95.29 |
| Young forest stands | 4548.45 | 1.24 | 0.02 | 2346 | 0.64 | 1.94 | 76.02 | 5.58 | 88.86 |
| Fallow land | 71.11 | 0.02 | 0.00 | 110 | 0.03 | 0.65 | 15.02 | 0.14 | 83.14 |
| Land suitable for afforestation | 1802.82 | 0.49 | 0.02 | 486 | 0.13 | 3.71 | 33.69 | 1.52 | 92.27 |
| Farmland | 7347.47 | 2.00 | 0.03 | 2459 | 0.67 | 2.99 | 97.49 | 9.11 | 88.73 |
| Water | 3145.15 | 0.86 | 0.10 | 129 | 0.04 | 24.38 | 70.28 | 4.24 | 87.62 |
| Construction | 1928.80 | 0.53 | 0.02 | 1853 | 0.51 | 1.04 | 68.48 | 3.28 | 84.59 |
| Unused land | 1562.81 | 0.43 | 0.07 | 775 | 0.21 | 2.02 | 43.09 | 1.80 | 89.32 |

Note: CA, class area; PLAND, percentage of landscape; LPI, largest patch index; NP, number of patches; PD, patch density; AREA_MN, mean patch area; LSI, landscape shape index; ED, edge density; AI, aggregation index

**Table S4** Landscape-level metrics used to analyze variation in *Monochamus alternatus* abundance in Ningshan County.

| Metric type | | | | | | | | |
| --- | --- | --- | --- | --- | --- | --- | --- | --- |
| Landscape metrics | TA/hm^2^ | NP | LPI/% | CONTAG | SPLIT | SHDI | SHEI | AI |
|  | 366711 | 8754 | 88.19 | 90.47 | 1.28 | 0.37 | 0.16 | 98.79 |
